# Extensive Metastatic Sarcomatoid Renal Cell Carcinoma Evaluated by ^18^F-FDG PET/CT: a Case Report and Review of Literature

**DOI:** 10.15586/jkcvhl.2018.99

**Published:** 2018-01-12

**Authors:** Dominique Fuser, Matthew L. Hedberg, Louis P. Dehner, Farrokh Dehdashti, Barry A. Siegel

**Affiliations:** 1 Division of Nuclear Medicine, Edward Mallinckrodt Institute of Radiology, Washington University School of Medicine, Saint Louis, MO, USA; 2 Department of Pathology and Immunology, Washington University School of Medicine, Saint Louis, MO, USA; 3 Alvin J. Siteman Cancer Center, Washington University School of Medicine, St Louis, MO, USA

**Keywords:** FDG PET/CT, metastasis, PET/CT, renal cell carcinoma, sarcomatoid

## Abstract

Sarcomatoid renal cell carcinoma (sRCC) is a highly aggressive form of dedifferentiated renal cell carcinoma. We report a 62-year-old man who presented with respiratory symptoms and a lung mass on chest computed tomography (CT). The patient underwent positron emission tomography/computed tomography (PET/CT) with ^18^F-fluorodeoxyglucose (^18^F-FDG) and was found to have extensive metastatic disease. Based on the history and imaging findings, there were possible primary malignancies, including bronchogenic carcinoma, melanoma, or an aggressive lymphoma. An excisional biopsy surprisingly revealed a high-grade sarcomatoid carcinoma with no evidence of differentiation, and immunohistochemical (IHC) studies showed that the tumor cells were positive for markers of genitourinary origin (PAX-8 and vimentin). The histologic and IHC results, along with multiple FDG-avid exophytic lesions in both kidneys, were considered diagnostic of sRCC. Here we have highlighted the potential role of ^18^F-FDG-PET-CT in patients with sRCC, discussed the diagnostic challenges, and presented a brief review.

## Introduction

The term “sarcomatoid renal cell carcinoma” (sRCC) was first used by Farrow et al. in 1968 to describe tumors that had progressed or transformed to a high grade, pleomorphic often spindle cell malignancy with a resemblance in most cases to pleomorphic undifferentiated sarcoma (1). Sarcomatoid dedifferentiation of a renal cell carcinoma (RCC) can occur with all histological subtypes. Since clear cell RCC is the most common subtype, approximately 80–90% of sRCCs are associated with this subtype (2). Sarcomatoid progression may occur in 10–20% of RCCs in patients with advanced disease and it is thought to be a major factor in RCC mortality (3, 4).

Positron emission tomography/computed tomography (PET/CT) with ^18^F-fluorodeoxyglucose (^18^F-FDG) has been shown to be a valuable tool in the detection of recurrent and metastatic disease in patients with typical RCC. However, characterization of the primary tumor can be difficult because of physiological excretion of FDG by the kidneys. Furthermore, well-differentiated RCCs often have only mildly increased FDG uptake that is similar to that of normal renal parenchyma (5–8). Since sRCC is more aggressive and has a higher metastatic potential than conventional RCC, ^18^F-FDG-PET/CT may play an important role in the management and follow-up of these cases. Review of the literature yielded only three previously reported cases of metastatic sRCC evaluated by ^18^F-FDG –PET/CT (9–11). In this report, we describe a case of sRCC in a patient in whom the initial staging ^18^F-FDG-PET/CT revealed widespread metastatic disease.

## Case Report

A 62-year-old man presented to his primary care physician with a dry, nonproductive cough with no hemoptysis for 6 weeks. He was initially diagnosed with atypical pneumonia and started on antibiotics, but his symptoms did not improve. The patient then presented to the emergency department at the Washington University Medical Center with worsening of cough, dyspnea on exertion, left rib pain, weight loss, and fatigue. A chest radiograph revealed a 9-cm middle mediastinal mass. Further workup with contrast-enhanced CT disclosed multifocal lesions, including an enhancing right lower lobe mass invading into the mediastinum, as well as multiple enhancing soft tissue nodules within the peritoneum, both adrenal glands and the pancreas. These findings were initially thought to represent a metastatic primary bronchogenic carcinoma.

**Figure 1.**
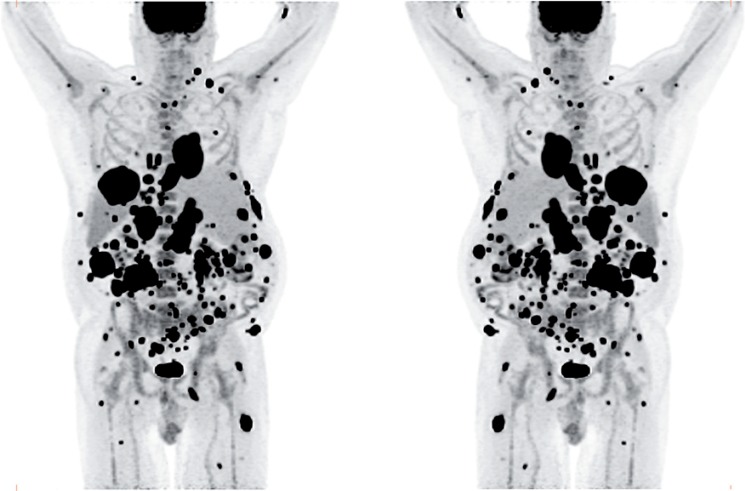
Maximum intensity projection (MIP) images in anterior and posterior views of the ^18^F-FDG PET/CT images showing multiple hypermetabolic foci of increased FDG uptake throughout the body, most likely representing widespread metastatic disease.

**Figure 2.**
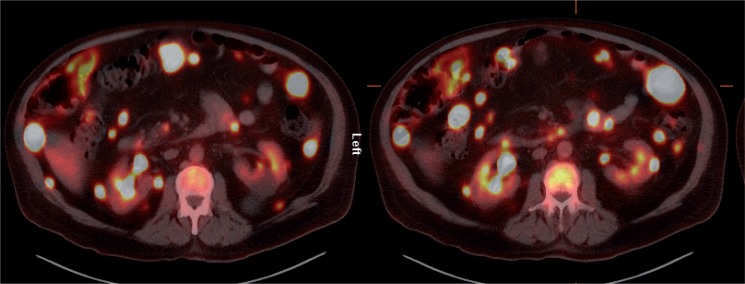
Axial ^18^F-FDG PET/CT fusion images. Foci of markedly increased ^18^F-FDG uptake are shown in the peritoneal cavity, and in exophytic lesions in the kidneys bilaterally.

The patient was referred for ^18^F-FDG-PET/CT the next day to further evaluate the disease extent. A whole-body PET/CT was performed 60 min after the intravenous injection of 629 MBq (17 mCi) ^18^F-FDG. The study showed multiple hypermetabolic foci throughout the body (Figure 1). There was a large soft tissue mass in the right lower lobe with increased ^18^F-FDG uptake; the maximum standardized uptake value (SUV_max_) of this lesion was 38.0. Also, there were multiple hypermetabolic nodules in the thyroid, gastric mucosa, pancreas, and both the adrenals. Both kidneys contained exophytic lesions with increased ^18^F-FDG uptake (Figure 2). A circumferential soft tissue mass in the esophagus was hypermetabolic as well. Additional FDG-avid nodules and masses were also found in the peritoneum and omentum; the largest of these had a SUV_max_ of 68.0. Intense FDG uptake was observed in multiple subcutaneous and intramuscular nodules throughout the body, some of which were thought to represent lymph nodes. Brain magnetic resonance imaging was performed on the same day, which revealed five metastatic lesions measuring up to 1.5 cm in the left frontal lobe, anterior right parietal lobe, and right temporal lobe. At this point, there were multiple candidate lesions for the primary malignancy, such as lung, esophagus, melanoma, or an aggressive lymphoma.

**Figure 3.**
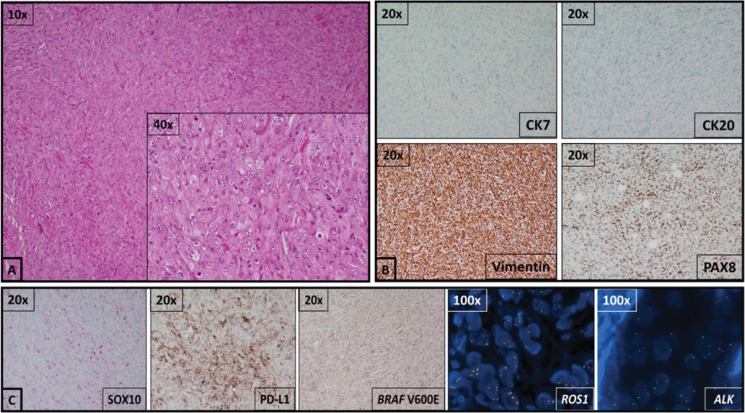
A diagnostic challenge. (A) On hematoxylin–eosin staining, this excisional biopsy demonstrates a lymph node that has been entirely replaced by a high-grade, eosinophilic, homogeneously sarcomatoid malignancy with no evidence of differentiation. There is a high degree of both cellular atypia, ranging from spindled cells to fried egg cells, and nuclear atypia, featuring vesicular chromatin, irregular nuclear borders, and prominent nucleoli. A portion of this tissue was sent for flow cytometry prior to microscopic analysis, which excluded a hematologic malignancy, as no significant lymphoid population was identified. (B) Immunostaining to identify the tissue of origin for this malignancy demonstrated tumor cell nonreactivity for cytokeratins 7 and 20, with reactivity for vimentin and PAX8. These immunohistochemical and morphological findings are most consistent with a high-grade sarcomatoid carcinoma of renal origin. (C) Interrogation of prognostic/predictive markers by immunohistochemistry demonstrated high PD-L1 expression levels and was negative for BRAF V600E hotspot mutation. Fluorescence In Situ Hybridization studies with break-apart probes were negative for rearrangements in ALK and ROS1, suggesting that this patient might benefit from treatment with pembrolizumab and would be less likely to benefit from treatment with crizotinib and dabrafenib.

An excisional biopsy of the right cervical lymph node was performed based on the ^18^F-FDG PET/CT findings. A portion of this tissue was sent for flow cytometry prior to microscopic analysis, to exclude a hematologic malignancy, and a standard lymphoma analysis was performed. Front and side scatter size gating found 0% of the cells to be blasts, and only 2% of the cells were identified as lymphocytes. Of these gated cells (lymphocytes), 0% were positive for the pan B-cell markers CD-19 and CD-20. Furthermore, no clonality assessed by kappa/lambda staining was identified. Of the gated cells, 95% were positive for the pan T-cell marker CD-3. This was consistent with entire replacement of the lymph node with tumor. The rare hematologic cells that were identified represented a small population of tumor infiltrating T-cells, a common phenomenon. The remaining portion was sent for histologic analysis, and was found to be a high-grade sarcomatoid carcinoma with no evidence of differentiation (Figure 3A). Immunostaining demonstrated the tumor cells were negative for both cytokeratin 7 and 20, and positive for vimentin and PAX-8, suggesting that the cancer in the sampled cervical lymph node was most likely a metastasis from a primary cancer of genitourinary origin. After correlation with the imaging studies, which identified bilateral, FDG-avid, exophytic lesions in the kidneys, a final diagnosis of sarcomatoid carcinoma of renal origin was rendered (Figure 3B). A detailed listing of the reagents used for histopathologic analysis of the tumor tissue is available on the journal’s website at: https://jkcvhl.com/index.php/jkcvhl/rt/suppFiles/99/0.

The patient underwent stereotactic radiosurgery to five brain lesions, and chemotherapy with carboplatin and paclitaxel protein-bound particles (ABRAXANE®) was immediately initiated. He did not tolerate the treatment well and CT showed progression of the disease. Second-line treatment with pembrolizumab was started. Unfortunately, a few days after the first cycle, the patient died.

## Discussion

sRCC is a highly malignant tumor that contains features similar to sarcomas, with spindle-like cells, high cellularity, and cellular atypia (4). Patients with sRCC have the worst prognosis among all patients with RCC, even among those with initially localized disease, with a median overall survival of 3 to 10 months (12). Preclinical and clinical data suggest susceptibility to cytotoxic agents and vascular endothelial growth factor-targeted therapies, but with poor response rates (13). A study by Joseph et al. has shown that sRCCs express PD-1/PD-L1 at a higher percentage than RCC without sarcomatoid differentiation, making sRCC patients good candidates for treatment with anti–PD-1/PD-L1 agents (14). Phase 1 data with atezolizumab, a PD-L1 antibody, showed early evidence of antitumor activity in sRCC, with an objective response rate of 22% (15). However, further studies are needed to establish the optimal combination of cytotoxic chemotherapy, targeted agents, and immunotherapy to improve outcomes in these patients.

The role of ^18^F-FDG-PET/CT for primary RCC is still controversial because of the renal excretion of ^18^F-FDG and the relatively low ^18^F-FDG uptake of many tumors (8). However, ^18^F-FDG PET/CT has shown to be effective for detection of suspected recurrent RCC (5, 8).

The use of ^18^F-FDG-PET/CT in the evaluation of sRCC has only been described in three prior case reports. Thambugala et al. described metastatic dissemination of a sRCC after nephrectomy (9). Conventional imaging of this patient with recurrent sRCC showed metastatic spread to para-aortic nodes, but ^18^F-FDG-PET/CT identified extensive local recurrence that involved the left renal bed and retroperitoneal soft tissues, as well as abdominal and mediastinal nodal and osseous metastases. Hyodo et al. reported a patient with sRCC in whom post-nephrectomy ^18^F-FDG-PET/CT demonstrated disseminated metastatic lesions in mediastinal lymph nodes, lungs, and the right infraspinatus muscle with intense ^18^F-FDG uptake (10).

Recently, Nadebaum et al. also reported a patient with recurrent sRCC, who had two subpopulations of disease with different imaging phenotypes on PET/CT with ^18^F-FDG and with the prostate-specific membrane antigen (PSMA) ligand, ^68^Ga-PSMA-HBED-CC. Based on the findings in this patient, they suggested that the sarcomatoid dedifferentiation is associated with a loss of PSMA expression and a concomitant upregulation of GLUT-1 transporter expression, increasing ^18^F-FDG avidity (11).

In all three prior reports, ^18^F-FDG-PET/CT was performed after nephrectomy to evaluate recurrent/metastatic disease and, therefore, evaluation of primary sRCC was not possible. Our patient had metastatic disease of unknown primary origin at the time of the PET/CT study. After the final diagnosis of sRCC, the renal lesions were recognized retrospectively as multiple hypermetabolic exophytic lesions bilaterally, with high ^18^F-FDG uptake relative to that of normal renal parenchyma. The findings in our patient also demonstrate the high ^18^F-FDG avidity of sRCC metastatic lesions, consistent with the findings of the three previously reported cases.

Histologically, this was a very high-grade lesion. The primitive phenotype of the cancer cells posed a diagnostic challenge in the setting of widespread disease and significant tumor burden in multiple organs, any of which could have been the primary tumor site. While the initial panel of immunostains suggested the primary lesion to be of urogenital origin, it also revealed a significant and unanticipated result, as portions of the tumor demonstrated positivity for SOX10, which is expressed predominantly in melanocytes, Schwann cells, and breast myoepithelial cells (16). Clinically, it is considered to be a relatively specific marker of melanomas, but it is also known to be positive in some malignant peripheral nerve sheath tumors as well as in some basal-like, triple-negative breast cancers, especially those with a metaplastic carcinoma component (17). Additional assessment of this lesion by MiTF and tyrosinase staining, both of which were negative, further supported that this tumor was not a melanoma. Rather, the tumor was most likely an sRCC aberrantly expressing primitive transcription factors known developmentally to be crucial in the specification of neural crest cells (16). To our knowledge, this is the first reported case of a SOX10-positive sRCC.

Given the unique cellular and molecular phenotypes observed in the excisional biopsy, it would have been interesting to sample additional lesions from this patient in order to assess whether or not a gradient of dedifferentiation and/or epithelial to mesenchymal transition exists between the primary tumors and the various deposits of metastatic disease, which demonstrated significant variation in the SUV_max_ on ^18^F-FDG-PET/CT.

## Conclusion

sRCC is a highly aggressive malignancy reported in some instances to overexpress GLUT-1 transporters, potentially resulting in high ^18^F-FDG avidity. Although the impact of ^18^F-FDG-PET/CT in sRCC has only been reported in three cases of recurrent disease, and must be further evaluated, the findings in our patient suggest that ^18^F-FDG-PET/CT is also potentially valuable for the evaluation of primary sRCC and initial staging for detection of metastatic lesions.

### Conflict of interest

The authors declare no potential conflicts of interest with respect to research, authorship, and/or publication of this article.
